# American Medical Association

**Published:** 1891-06

**Authors:** 


### Society Notes.

### AMERICAN MEDICAL ASSOCIATION.

Mhe fifty-second annual meeting of the American Medical Association was held in Washington, D. C., May 5th, 6th, 7th and 8th, 1891.

At 10:30 a. m. the first general session of the Association was called to order by Dr. D. C. Patterson, Chairman of the Committee of Arrangements, in Albaugh's Opera House.

Rev. Dr. S. M. Newman opened with prayer, followed in an address of welcome by the Hon. John W. Ross, one of the District Commissoners.

Mr. Ross said he felt honored in being called upon to welcome such a body, as it was a representative body of the profession of a continent, the like of which could be found in no other land.

He then spoke of the importance of a body like this in securing proper legislation on medical matters, and particularly of professional secrecy. Many States had already passed laws recognizing the sacred relations of physician and patient. The District of Columbia still clung to the old common law, and he suggested that Congress be asked to pass a law so that the veil of secrecy might be drawn over the confidences of practitioner and patient.

The Hyppocratic Oath, although the conception of a Pagan moralist was sublime in its idea of a physician, but the speaker thought that the modern Christian physician should and did exceed that idea.

Mr. Ross suggested Washington as the permanent home for the Association, because Washington belonged to all Americans and he could think of no place better fitted for a permanent abiding place.

The ex-presidents of the Association were then invited upon the platform, and Dr. Patterson welcomed the members in a short address, after which he introduced the President of the Association, Dr. W. T. Briggs, of Nashville, Tenn. The Doctor said :

" Medicine is the connecting link between science and philanthrophy, and that the gentlemen present were there for the sole purpose of promoting science and the good of humanity. That this Association was the outgrowth of the need felt of co-operation among the physicians. That the Association had brought about, to a very large extent, the unification of the profession through its Code of Ethics, which had done so much to elevate the profession in public estimation, and to form a barrier between the sheep and the goats.

"Medical education had always been one of the chief aims of the Association, and had resulted in great good as shown by the gradual raising of the standard of the various colleges. That the graduates of our medical schools were the peer of all others and exceeded all in surgery."

As nearly all colleges had accepted the suggestions of the College Association, Dr. Briggs thought that it would be proper and right that the privilege of membership be refused to all who hereafter be graduated from or be professors in colleges not coming up to the standard.

The President said that there was too much routine work in a business way done and not enough scientific, and suggested that a committee of two from each State be appointed to look after the ordinary business of the Association, and report at the general session daily, so that the time of the general session could be devoted to scientific discussions. The Doctor also thought that a section of original research should be formed, or that prizes should be offered for essays on subjects of original thought and research. Another suggestion of the President, and a good one we think, was that, to make the Journal what it should be, an editor of acknowledged worth and ability must be had, and that he should be paid a handsome salary of from $10,000 to $15,000 per year.

After the President's address, a vote of thanks was offered him and a committee appointed to look into the suggestions offered.

The report of the Trustees of the Journal was then read, but their recommendation of the removal of it to Washington was lost by an overwhelming majority.

Dr. Comegys, of Cincinnati, moved that a committee be appointed to consider the advisability of petitioning Congress to create a new cabinet officer of public health.

Dr. N. S. Davis announced that there would be a meeting to establish a National Medical Temperance Society.

After the announcement of the Committee on Nominations, the general session adjourned for the day.

SECOND DAY.

The session was called to order by Dr. Patterson, the prayer being made by Rev. Father Richards. Dr. E. L. Sharley followed with an address in general medicine on The Difference Between Life and Death.

To give an accurate account of this paper would take more space than we can allow at present. It was one of the best addresses of the meeting.

The Committee on State Medicine was abolished, and their work placed in the hands of the Section on State Medicine.

It was suggested and left to a committee of five to confer with the societies, that all State and District Societies having a membership of over one hundred be recognized as branches of the American Medical Association, and their members have all the rights and privileges of delegtes.a

A communication was then read from the Medical Society of West Virginia, asking for counsel in regard to the treatment by the body of the profession of surgeons in the employ of railways, and for a decision of the question as to how far their conduct was in conflict with the Code of Ethics. It was claimed that railway surgeons give their services to rich corporations, taking passes in lieu of fees or other emolument, a proceeding derogatory to the dignity of the professisn, and also that they always assumed entire and sole charge of every case of railway injury, to the prejudice of the family physician or other attendant who may have been first called. A committee of one from each State to investigate this matter was then appointed. Session then adjourned.

THIRD DAY.

The general session was opened with prayer by Rev. Dr. Bartlet.

The Trustees reported that the Journal was doing well, but no editor had yet been appointed, and that the place of publication would be Chicago.

The Nominating Committee reported as follows:

President—H. O. Marcy, of Boston.
First Vice President—W. P. King, of Missouri.
Second Vice President—Henry Palmer, of Wisconsin.
Third Vice President—W. E. Davis, of Alabama.
Fourth Vice President—W. E. Taylor, of California.
Secretary—W. B. Atkinson, of Philadelphia.
Treasurer—R. J. Dunglison, of Philadelphia.
Librarian—G. W. Webster, of Chicago.
Trustees—W. W. Dawson, W. W. Potter and J. H. Rauch.

The next meeting will be held in Detroit, on the first Tuesday in June, 1892.

The address on Surgery was delivered by Dr. J. M. Mathews, of Louisville, on the subject of Stricture of the Rectum.

The session then adjouned.

